# Notes on Preparations for the Sick

**Published:** 1898-12

**Authors:** 


### Notes on Preparations for the Sick.

**Protargol.**—FRIEDR. BAYER & Co., Elberfeld.—This is a smooth yellowish powder easily soluble in water to the extent of 50 per cent., forming a brownish solution. It contains 8 per cent. of silver in proteid combination, so that it is very unirritating locally, and can be used for the urethra, conjunctiva, &c., as a lotion of the strength 1 to 5 grains to the ounce. We have employed it in several cases for the urethra, and in one case for the bladder, with success. In one case, however, the patient's urethritis seemed to be increased. Favourable reports of its action are numerous, including its effect in acute conjunctivitis.

**Trommer's Extract of Malt with Cascara Sagrada.** — The Trommer Extract of Malt Company, Fremont, Ohio.—This combination is a very useful one for weakly ill-nourished patients with constipation. The malt extract makes an exceedingly good vehicle for the bitter extract, which has a stimulating effect on the mucous membrane as well as on the muscular fibres of the intestinal walls. One tablespoonful contains the active properties of 30 grains of Cascara Sagrada; this dose may be given three times a day after meals, but a smaller dose will commonly suffice.

---

**Lacto-Glycose.** — Mellin's Food, Ltd., London.—This is a dry powder prepared from Mellin's food and sterilised cow's milk. It does not contain starch, and the caseine of the milk is broken up mechanically, so that the combination is easy of digestion and may be given to infants under three months as well as to delicate children and invalids of any age. It forms a complete food, highly nutritious, palatable, and easily assimilated.

---

**Medical Izal. Izal Ointment. Izal Cream. Izal Lint. Izal Wool. Izal Gauze. Izal Medical Soap. Izal Soap. Izal Soft Soap. Izal Perles.** — Newton, Chambers & Co., Ld., Thorncliffe, Sheffield.—So long ago as December, 1895, we called attention to the virtues of Izal, one of the numerous coal-tar derivatives. Supplementing Dr. Klein's favourable opinion, to which we then referred, comes that of Prof. Sheridan Delépine, who speaks of "the immense advantages which Izal possesses over carbolic acid in many directions." In Notter and Firth's well-known *Theory and Practice of Hygiene* strong approval is given to Izal as a trustworthy disinfectant. Surgical authorities also speak well of it as a dressing for wounds. We have received the preparations which are named at the head of this notice, and the list shows to what a variety of uses Izal is now put. The manufacturers point with pride to the fact that its germicidal power has obtained Government recognition, and that they have received orders from the War Office, from Her Majesty's Office of Works, and from the Admiralty. For ordinary disinfectant purposes it is sent out in bottles or in perforated tins. Other uses of it are sufficiently indicated by the names of the preparations. The **Perles** are recommended to be given in enteric fever and obstinate diarrhœa.

---

**"Sirol" Antiseptic Tooth Paste.**—Gustav Hermanni, Jun., London.—We are not told of what this consists, but are informed that "its ingredients are such as would serve as a neutralising agent to the acid secretions of the mouth, and its aromatic constituents act as an antiseptic." Any way it is pleasant to use, and it is conveniently supplied in collapsible tubes.

